# Contributions to the Analysis and Synthesis of Pseudo-Plastic Processes

**Published:** 1845-07

**Authors:** 


Art. V.

*Zur Analysis und Synthesis der pseudo-plastischen Prozesse im Allgemeinen und einiger im Besonderen.* Von Dr. Gustav Zimmermann.—*Berlin.*
*Contributions to the Analysis and Synthesis of Pseudo-plastic Processes.* By Dr. G. Zimmermann, Assistant-Surgeon in the Prussian Army.—*Berlin,* 1844. 8vo, pp. 356.

These analytical contributions to the pathology of the blood and of inflammation exhibit considerable research and industry on the part of the author, and are of importance and interest, as our readers will see.

The author first lays down a theory; he then details cases of thoracic or ophthalmic inflammation investigated with reference to the theory; and next explains the application of the facts observed in the theory detailed. Our principal object will be to record the facts, leaving their theoretical and practical application to our readers. It is but right, however, to introduce Dr. Zimmermann's facts by a short exposition of his views.

There are two modes in which elementary bodies combine, the chemical and the organic; and these two modes divide the transformations of matter which result from their combinations with each other into two distinct classes. The chemical transformations are regulated by a stoichiometric law (stoicheion, elementum,) or law of atomic combination, the atoms uniting in the proportion of one to one, two to two, &c.

The organic transformations are not regulated by this law, and the atoms of organic bodies do not unite according to a fixed ratio. The arrangements of their atoms must be very different from that in inorganic bodies, since urea presents characteristics differing altogether from cyanate of ammonia although the two bodies are identical in atomic composition.

Proteine is the representative and basis of animal compounds, as albumen, fibrine, &c. Caloric, oxygen, and water are the means whereby the chemical mutations in proteine are commenced and carried on. The oxygen of the atmosphere is the representative of putrefaction; it reduces the organic to the inorganic.

Whilst we recognize a power of organic affinity in each atom of organic matter, we recognize also a vital power in each congeries of organic atoms, brought together and arranged after a certain form and a definite idea, and for a distinct object. The atoms thus united into a whole constitute a cell, and the cell, as a whole, combines with each other the atoms of which it is composed. In this respect is its difference from proteine compounds in which the mutual action is between the atoms themselves. Together with the vital composition and recomposition of the atoms there is a change in the cell itself; the weaker the affinities are between the former, the weaker is the vital power in the latter, by which its constituent atoms are united.

Such of our readers as have made themselves acquainted with the views of Schultz will now know where they are. Every cell is a microcosm: it has a beginning, a continuation, a decline; during its course it takes up new atoms and casts off effete, until its course is ended. Cell-life, however, is not an independent life; a combination of cells after a definite idea and with a distinct object constitutes a tissue. Here union is force; united they perform higher and more important functions. Still, however, each cell has its individual life ; it is born, grows, ripens, becomes old, disappears, and in the place of the cell gone, a new cell comes, to run through the same cycle. A union of several tissues constitutes an organ ; a union of several organs constitutes an organism, or individual. The united powers of the organism constitute its individual vital power; the whole of its functions constitute its life. So that the vital power of an organism is in proportion to the vital powers of the organs, entering into its composition; the vital powers of the organs depend upon those of the tissues, those of the tissues depend upon those of the cells, and those of the cells depend upon the force of affinities of the organic atoms entering into their composition. Our readers may go to the last step if they please, and say that the force of these latter depend on the energy of the organic force, but for the purposes of the theory we had better stop at the cells.

To get then at the root of the matter, and know clearly the origin of morbid processes, we must know the course of cell-life. We must examine the mode of their progression or development, and of their regression or decline; and we must learn how external injurious agencies (*schädlichen potenzen,*) act on them, and so change a healthy vital process of the organism into a morbid. In short we must ascertain the bio-chemical relations of their constituent atoms.

*The normal progressive metamorphosis of the cells.* The chyle, the product of organized matter, is the source of nutriment to the cells. Its two principal constituents are fat and albumen. The granules of the lymph are formed out of the fat, and their containing vesicles out of the albumen, (Vide British and Foreign Medical Review, vol. XVI, p. 212,) but which gradually change after their formation into casein or globulin. Having in the article on the doctrines of Schultz (to which we have just referred) set very clearly forth the life and adventures of these vesicles, we need not here repeat our story. It will be sufficient to say that being cells themselves they carry oxygen to the cells of the tissues, where it unites with carbon, forming carbonic acid, with which being laden they return to the lungs, there to give it out and take in a fresh cargo of oxygen. They are gasiferous cells, but not cibiferous. What then is the nutritious material in the blood? The albuminate of soda. Each tissue abstracts electively the albumen to form its new cells, but in forming the new cells a new arrangement of its atoms takes place, it is transformed, and is no longer albumen. Each tissue has in itself the power to appropriate the albumen to itself by a special transformation; thus muscle forms it into muscle, nerve into nerve, &c. It is generally held by physiologists that the fibrine of the blood is the source of the new matter deposited in the tissues, and it is in holding a contrary opinion that Dr. Zimmermann's theory essentially differs from others. According to his views, the fibrin is the product of the regressive metamorphosis of the cells, and that therefore it is a purely excrementitial product. His proofs are the following : There is no fibrine in chyme, and very little in the chyle, and, what is remarkable, much less in the chyle of carnivorous animals than in herbivorous, as horses and sheep. Neither by nutriment of the most varied quality nor by hunger is the small quantity in the chyle of horses diminished, but on the contrary, rather increased, if we can rely on the experiments of Tiedemann and Gmelin, who concluded that the fibrine must get into the chyle through the lymphatics. Since, then, there is no fibrine in the chyme off carnivorous animals, while it constitutes so large a portion of their food, the object of digestion must be the transformation of fibrine into albumen. The younger the animal eaten, the more facile the digestion of its fibre, because the nearer the latter approaches in composition to albumen. Further, the blood of carnivoræ contains less of fibrine than the blood of herbivoræ. The lymph, as Dr. Zimmermann shows, is loaded with fibrine, which would not be the case if fibrine were the nutritive constituent of the blood. Another proof quoted by him is the result of the experiments instituted by Magendie, Nasse, and others, who, having transfused blood deprived of its fibrine into an animal, found that after being circulated awhile it contained fibrine and was coagulable; a fact not to be explained by the opinions of Schultz, Simon, and others, as to the origin of fibrine. Dr. Zimmermann further argues, that venous blood contains more fibrine than arterial, although he acknowledges that in this point further experiments are required.

These are the principal proofs brought forward by the author in support of his doctrine. It is true he enters into details we have not noticed, to show that other opinions as to the origin and use of the fibrinous constituent of the blood are wrong: but granting this, it is no proof he is right. In a subsequent section it will be seen our author assumes fibrine to be an excrementitial product of the muscles.

*The normal regressive metamorphosis of the cells.* In every moment of life, in every movement whether voluntary or involuntary, in every act of every organ, in every sensation, idea, or perception, there is necessarily a change in the relations of the organic atoms constituting the basic cells of the organism. The latter are capable of performing their proper functions so long as these changes do not induce such chemical transformations as to destroy the vital force which unites their atoms; but when this metamorphosis is perfected, the effete cells must be excreted and give place to others, otherwise a morbid state of the organ is set up. The oxygen taken up by the blood-vesicles, and brought through the capillaries to the basic cells, is the principal agent in effecting these changes. Fibrine is the product of the regressive metamorphosis of the muscles, and uniting with oxygen forms the normal products excreted by the skin and kidneys. Increased muscular action increases the quantity of fibrine in the blood, and of lactic acid in the urine, while Dr. Zimmermann engages to produce pathological observations in proof that the fibrine in the blood is resolved into inorganic products, as urea, &c. He argues that the experiments of Prevost and Dumas, who having extirpated the kidneys of animals found urea in the blood, to prove that the formation of this and the other urinary constituents is not due to the action of the kidneys themselves, but rather that they are merely the emunctories through which substances already formed during vital processes are eliminated from the circulation. Previously to giving his own theory in this matter, our author combats those of Prevost and Dumas, Tiedemann and Gmelin, Müller, Simon, Berzelius, and Schultz, and shows that they must all be wrong, and then by special pleading shows that he is right. Adopting Liebig's views as to the relations of the compounds of proteine to oxygen, he illustrates his scheme of the metamorphosis of fibrine by the following:

FIBRINE.

| Urate of ammonia | | | Choleic acid. |
|---|---|---|---|
| | Sulphur: Phosphate of lime | Phosphorus | |
| Urea: Carbonic acid | | | Carbonic acid. Carbonate of ammonia. |
| | Sulphuric acid. | Phosphoric acid | |
| | Sulphate | Phosphate. | |

*Pathological corollaries.* This section is introduced with the theoretical remarks usually made by systematic authors, the intent of which is to show that by various injurious agencies acting on the cells during their progressive and regressive metamorphoses, a pseudoplasma is necessarily formed in the blood, represented by pseudo-fibrine, pseudo-albumen, and pseudo-globuline, the specific differences being due to variation in their atomic constitution. It is possible, for example, that hydrogen may be in a less, and oxygen in a greater proportion in the fibrine of rheumatism: or that in the fibrine of erysipelas, the carbon may be increased and the oxygen diminished. In contagious diseases, a metamorphosis of the plasma into a pseudo-plasma is effected by the miasma introduced into the circulation assimilating the atomic constituents to its own composition. Dr. Zimmermann steadily carries out the application of his doctrine to general pathology, and explains the variations observed in the nature and seat of diseases, and the doctrine of crises, and critical days, advocating hepta-periodicity. The following is his own summary, which we give to avoid the necessity of our entering further into details.

| | |
|---|---|
| "1. A pseudo-plasma is formed. | 2. There is a locus minoris resistentiæ. |
| *a.* A portion is deposited in an organ. | *a.* Through the deposition of the pseudo-plasma it becomes a morbid spot. |
| *b.* The other portion remains in the blood, and is decomposed by the action of the oxygen derived from the atmosphere. | *b.* The phenomena of reaction, with beneficial or injurious results. |
| *c.* And consequently, from the commencement of the disease, anomalous products appear in the urine, perspiration, &c. | *c.* In favorable cases, the pseudo-plasma and other constituents of the organ undergo such change, that either they are excreted directly, or are reabsorbed into the blood. This is the local crisis. |
| *d.* The anomalous condition of the excretions continues so long as pseudo-plasma remains in the blood. | *d.* The matter reabsorbed is again poured forth through the excreting organs. |
| *e.* All abnormal conditions of the excretions happening during the course of a disease, are to be considered as critical phenomena, in case they correspond to a materia peccans. | *e.* The abnormal constituents of the urine, perspiration, &c. increase and continue so long as a pseudo-plasma remains in the blood. |

"The general critical phenomena, as in the urine, cease on particular days, and have a type of three days and a half." (p. 99.)

*Contributions to special pathology and therapeutics.* The first of these is a paper on milky serum. Of 17 cases which he observed out of 50 persons bled, 12 were diseases of the eye; 2, thoracic inflammation; 1 cerebral congestion, and 1 a case of hypertrophy of the liver, spleen, &c.

The specific gravity of the serum varied from 1027 to 1036, and 1000 grains contained from 96 to 112 grains of solid matter. It was of a rather thick consistence, sometimes greenish, sometimes yellow, the clot appearing through it of a violet colour, and in only three cases was there a buffy coat. A general recapitulation of all the observed cases of this kind is given, and an account of his own analysis, from which we gather nothing satisfactory. Next comes a paper on the existence of fibrinous serum; and another on milky serum with reference to the milk-secretion of wet nurses and pregnant females, both of which being polemical, we pass them over to notice his remarks

*On coagulable urine.* The discovery by Dr. Bright has led to numerous researches on the constitution of the urinary secretion with special reference to the presence of albumen. It has been observed in the urine of persons suffering from widely different diseases, as scarlatina, and the dropsy following thereon, meningitis, bronchitis, pneumonia, peritonitis, diabetes, diseases of the spinal cord, of the heart; and has even been observed in persons having no manifest disease whatever.

Fibrine is, however, sometimes present in the urine. Brandis found that of a person affected with scarlet fever to coagulate on cooling. Nasse knew a catholic priest who passed, particularly during the night, a large quantity of whitish urine that coagulated spontaneously in from ten to fifteen minutes after leaving the bladder, and often indeed coagulated in the bladder itself. The patient experienced no debility. On analysis, the urine was found to contain a large quantity of fibrine, but no blood-globules. There were also prismatic crystals in the urine which were found to be triple phosphates.

Dr. Zimmermann has had patients whose urine coagulated by heat, but on applying the proper tests it was found to contain another compound of proteine than albumen. He thus describes the urine in which he detected fibrine. From patients with inflammatory affections it was saturated, acid, and urinous in smell; in other cases it was pale, cloudy, of a faint sweetish smell, and nearly or altogether alkaline. Sometimes it contained, besides fibrine, amorphous urate of ammonia or uric acid. Dr. Zimmermann observed fibrinous urine in endocarditis, pleuritis, pneumonia, inflammatory catarrh, rheumatic ophthalmia, periostitis of the occiput, and erysipelas of the face. In five of these cases, two being rheumatic ophthalmia, and three thoracic inflammation, the blood contained molecular fibrine. In these cases Dr. Zimmermann observes that the kidneys were quite healthy. The fibrine appeared in the urine simply as an excretion, sufficient oxygen not having been taken up to decompose it into its organic forms. Our author thinks that in many cases of coagulable urine, the coagulation will be found to be due to the presence of fibrine quite as often as of albumen. It will be found probably in cases where the cutaneous function is suppressed, as happened in the examples he analysed, and also be accompanied with fibrinous serum.

*On the action of the salts which dissolve fibrine.* We pass over two sections on the urinary salts to this subject, Dr. Zimmermann having been induced to direct his attention to it by the peculiar doctrine he holds as to the origin of the fibrine and of the pseudo-plasma, or materia peccans. The salts having this property are the carbonate, borate, and phosphate of soda, and the nitrate, sulphate, acetate, and hydriodate of potass. Their action on the fibrine is not purely chemical, as they cause no change when brought into relations with that compound. The salts may be seen under the microscope to separate unchanged. They exercise a contractile influence on the capillaries, and also on the blood-vesicles themselves, deepening their colour, and exciting the secretion of carbonic acid, plainly in consequence of a more active oxydation of the organic ferruginous compound the vesicles contain. All these salts act on the whole mass of the blood into which they are absorbed. Dr. Zimmermann had a striking proof of this. He gave two drachms of nitre daily for seven successive days, to a soldier labouring under arteritis, and having occasion to bleed him on the seventh, examined the serum. It presented a bluish white appearance, and deposited on the inner surface of the stone-jug in which it was contained such a quantity of the salt, that it might have been collected by a scruple at a time. The atrophy which the continued use of these salts induces proves that, while they promote the regressive, they interrupt the progressive metamorphosis of the tissues.

*The condition of the blood and urinary secretion in thoracic inflammation.* We pass over some researches instituted to determine the proportion of the various constituents of the blood, and a critique on the method of analysis practised by Andral and Gavarret. Dr. Zimmermann details his own method which is as follows.

*How to conduct a pathological analysis of the blood.* 1. A glass containing exactly 1000 grains of water is filled with blood directly from the vein; a glass stopper fitting hermetically is then inserted, (care being taken to remove all bubbles,) and the specific gravity of the blood ascertained. By continually shaking it the blood is kept fluid; it is then poured into a porcelain vessel, and evaporated, care being taken to wash the glass out with distilled water, and add the latter to the other. The quantity of solid residue is reduced to the proportions in 1000 grains of blood. 2. A glass two and a half ounce measure is filled with the blood immediately after the preceding, covered, and set aside for eighteen hours. The clot is then carefully separated from the serum, and the weight of both ascertained. 3. The clot is now to be pressed through a linen cloth four inches square, and first the fibrine, then the fluid cruor collected. Each is thoroughly washed, freed from moisture, and weighed; they are then carefully dried and again weighed. The results are to be reduced to the proportion in 1000 grains of blood. 4. The serum and clot are to be placed in a glass containing 360 grains of distilled water, and their specific gravity noted, then 250 grains of each at the same temperature, are to be dried so long as they lose weight; the weight of the residuum is then to be reduced to the proportion in 1000 grains. There is a loss of weight by pressure through the linen, but as this will happen in the same proportion in all instances the general results are correct.

Dr. Zimmermann observes that this method will be more suitable to the practical physician than Andral and Gavarret's, because of its greater simplicity and easier performance, while the results are quite as correct as by the latter method.

*Condition of the blood in pneumonia and pleuritis.* Dr. Zimmermann details four cases in which he made a daily examination of the urine, and also examined the blood after the method described when it was necessary to practise venesection. The fourth case (pneumonia) was complicated with enteritis, and is worthy of notice from this circumstance, that although he learnt by the stethoscope that the left lung was almost completely hepatized at an early period, there was neither cough, pain, nor any rational sign of thoracic disease until the seventh day. The patient died on the ninth, and the autopsy showed the stethoscopic diagnosis to have been correct. A very elaborate analysis and comparison of the various phenomena observed, and especially the mutations in the urine is given, and also a less elaborate statement of the condition of the blood in seven other cases of pneumonia and pleuritis.

In six cases in which the blood had the buffy coat, ten bleedings were practised. In six other cases, there was no buffy coat, and eight bleedings were practised. The following is the summary of each, and the general average of the whole. The quantity of blood examined in each case was 1000 grains.

*Blood presenting the buffy coat.*

| The whole blood. | | Serum. | Clot. |
|---|---|---|---|
| Specific gravity . . . | 1049·8 | 1027·5 | 1079·4 |
| Solid contents . . . | 203·3 | 93·3 | 286·0 |
| Water . . . . | 796·7 | 906·7 | 714·0 |

Moist fibrine, 20·6 grs.; dry fibrine, 6·1 grs.; proportion of clot to serum, 1·84 to 1.

*Blood not presenting the buffy coat.*

| The blood generally. | | Serum. | Clot. |
|---|---|---|---|
| Specific gravity . . . | 1050·8 | 1027·5 | 1084·5 |
| Solid contents . . . | 205· | 93·6 | 299·3 |
| Water . . . . | 795· | 906·4 | 700·7 |

Moist fibrine, 11·8 grs.; dry fibrine, 3·58 grs.; proportion of clot to serum, 1·76 to 1.

The twelve cases estimated according to the method of Andral and Gavarret gave the following results:

| | Water. | Residuum. | Fibrine. | Blood-vesicles. | Residuum of serum. |
|---|---|---|---|---|---|
| The mean of the twelve . | 796·2 | 203·9 | 4·75 | 118·1 | 80·85 |
| Mean of Andral and Gavarret's analyses (58 cases) | 799· | 201· | 7·3 | 114·1 | 81·0 |

Simon of Berlin analysed the blood of two healthy persons, and of four suffering from pneumonia with the following mean results; his method differing from both the preceding.

| | Water. | Residuum. | Fibrine. | Fat. | Albumen. | Globulin. | Hæmatine. | Salts, &c. |
|---|---|---|---|---|---|---|---|---|
| Healthy blood . | 795·27 | 204·72 | 2·10 | 2·34 | 76·60 | 103·02 | 0·209 | 12·06 |
| Blood in pneumonia | 810·73 | 189·26 | 6·06 | 2·84 | 106·12 | 59·13 | 2·53 | 10·01 |

The difference between the healthy and diseased blood can be readily estimated by our readers.

*Theory of inflammatory blood.* Dr. Zimmermann proceeds to theorize on his facts and to apply the variations in the constituent elements of the blood to the explanation of thoracic inflammation. These affections are most frequent in winter with a north or north-west wind, when the temperature is low, and the weather fine and dry. From the absence of moisture, and of the gases contained in the air during the heats of summer, more oxygen is taken into the lungs at each inspiration. The consequence is a more rapid evolution of the blood-vesicles and a consequent diminution in their number. Further, the congestion of the lungs in inflammation and the depletion practised remedially will diminish their relative proportion. They also exhibit a peculiar facility of uniting and adhering to each other in inflammatory blood, such as is not observed in the normal condition. This characteristic appears to depend on certain transparent irregular masses of matter attached to the margins of the vesicles, and in which some granules are to be detected. The saline constituents are diminished, and the albuminate of soda increased, the increase of the latter being dependent, according to Dr. Zimmermann, on the increased quantity of food taken in winter to maintain the animal heat, and the consequent reaction of the oxygen in the organic atoms thus introduced to keep up the metamorphosis of the tissues. The skin being subject to a lower temperature in winter, its excreting functions are less active, and less decomposed fibrine is thrown off through this emunctory. In ordinary circumstances, the kidneys supply the deficiency, but when an individual has taken cold, which usually occurs when heated after exercise, and when there is necessarily an unusual quantity of fibrine in the blood, that substance being the moult or product of the regressive metamorphosis of the muscular fibre, then the functions of the skin are altogether abolished, and the kidneys being unequal to the additional duty imposed on them, the fibrine accumulates, and loading the capillaries gives rise to congestion of the lungs. This congestion by diminishing the supply of oxygen still further aggravates the original disease, and thus the pseudo-plasma is formed in the lungs, or if not in them in the *locus minoris resistentiæ.*

*Explanation of the changes in the physical characters of inflammatory blood.* Inflammatory, and particularly buffy blood, is thinner, brighter coloured, and produces more foam as it flows into the vessel, than normal blood. The fluidity is dependent on the greater amount of fibrin and serum it contains. The albumen and fibrin both take up a larger quantity of water to hold them in solution. The brighter colour depends on the higher degree of oxydation attained by the hematine. The causes of the slower coagulation of buffy blood are,—1, the increase in the quantity of fibrin; 2, the diminution of the number of blood-vesicles, and their tendency to adhere to each other; 3, some peculiar vital properties of the fibrin itself. The blood-vesicles, Dr. Zimmermann thinks, act as points of crystallization, just as a straw thrown into freezing water accelerates the formation of ice. The same causes which retard the coagulation of the blood, facilitate the formation of the buffy coat. These same causes also operate in the formation of the buffy coat, and cause a difference in the specific gravity of different portions of the clot. In coagulated healthy blood, the lowest portion is the heaviest, the heavier vesicles sinking to the bottom. Dr. Zimmermann instituted inquiries on this point with the following results. In 1000 grains of the upper portion, there were 320 grains solid contents, the specific gravity being 1090; in 1000 grains of the lower, 368 grains solid contents, and specific gravity 1104. With this tendency of the blood-vesicles to sink, there is an opposite tendency of the fibrine to rise, and thus form the buffy layer or coat.

In 1000 grains of the upper and under portions of various specimens of blood examined, the proportions of fibrine was found as follows:

| | Fibrine in Upper portion. | Fibrine in Under portion. |
|---|---|---|
| Blood of a healthy person | 7·3 grs. | 2·7 |
| In a case of congestion of the head | 6·5 .. | 2·3 |
| bilious pleurisy | 9·2 .. | 3·5 |
| pneumonia | 14·8 .. | 3·3 |
| nephritis | 13·7 .. | 6·3 |

But although the formation of the buffy coat proves the existence of an abnormal quantity of fibrine in the blood, it is not diagnostic of pneumonia. On the other hand, the absence of the buff is no proof that the fibrine is not in excess. It has been already shown that in twelve cases of thoracic inflammation, the buffy coat was observed in six only. The reasons why this is so, are adduced by Dr. Zimmermann as follows. It depends firstly, on the amount of blood-vesicles in the blood previously to the attack of inflammation; secondly, on the quantity of accumulated fibrine; and thirdly, on the extent to which the effusion of plasma consequent on the congestion takes place, and on the number of blood-vesicles accumulated in the capillaries. If the buffy coat is not formed at the first bleeding, it often is at the second, especially in those cases in which the inflammation is peracute, and the skin dry. The increase of the fibrine consequent on the interruption of the cutaneous and pulmonary functions, the using up of the blood-vesicles, and the greater fluidity of the plasma facilitate the formation of the buffy coat. It often happens that when the blood at the same venesection is received into successive vessels, a buffy coat forms in some and not in others, but most frequently the latter predominates, as for example, when from ten to twelve ounces are received into four or five cups. The reason of this is not evident, nor are there any circumstances in Dr. Zimmermann's experience that elucidate this point. He does not find the pulse to be a guide. In pneumonia, where a buffy coat was formed at the first bleeding, he has found the pulse quick, frequent, soft, and intermittent; in erysipelas, where also this coat was seen, the pulse was strong, frequent, round, and normal. On the contrary, in pleuritis, with a quick, rather round and hard pulse, no buffy coat was found.

*Pathology of thoracic inflammation.* Dr. Zimmermann devotes several pages to a discussion of this subject, and applies the theory of the formation of pseudo-plasma already detailed to its elucidation. Amongst the predisposing causes, he considers first the condition of the blood itself as it is observed in those predisposed, namely, an excessive richness of the blood, and great vital activity in the primary cells of the tissue, or in other words, a plethoric condition. Amongst the exciting causes, is the interruption by cold of the action of those organs whose function it is to excrete the rapidly accumulating fibrine namely, the skin and kidneys, and which facilitates the formation of the materia peccans of the humorists, or the pseudo-plasma as he prefers to call it. The lungs are constituted the *locus minoris resistentiæ* because of the greater activity of their tissues in youth, the age at which inflammation of their parenchyma or serous coverings most frequently occurs. Further, the transformation of the atoms constituting fibrine being prevented by the interruption of the cutaneous function, the access of oxygen being there barred, the lungs take up a vicarious action, and so become the seat of the pseudo-plasma. The first action of this on the pulmonary capillaries is that of an irritant, causing their contraction. Reaction is then set up; the circulating fluid presses forward; the capillaries are dilated, then congested, and ultimately effusion of the pseudo-plasma into the surrounding parenchyma takes place; or where the capillaries give way to the pressure from behind, blood itself is effused. With this effusion the proper phenomena of inflammation cease, and a new vital action directed to the effused fluid is set up, constituting the completion of the first crisis of the disease. These crises have a three and a half day type (a lunar week), the normal course of a pneumonia or pleuritis occupying eight lunar weeks or twenty-eight days. In fourteen days, the series of morbid changes arrive at their climax; they then begin to decline, and in fourteen more are ended. He has observed a similar periodicity in erysipelas, and thinks the cause is to be sought for in the metamorphosis of the pseudo-plasma and of the cells in the blood and nervous system. We cannot follow our author through his explanation of the pain, cough, dyspnea, headache, increased frequency of the circulation and respiration, and the other symptoms of thoracic inflammation; to all these he applies his own doctrines, or those generally received with great ingenuity. With equal ingenuity he discusses the operation of the remedies usually found most successful in treatment, and giving their biochemical and physiological action with great minuteness. Bleeding, with nitrate of potass and tartar emetic, or the two latter without bleeding, and also acetate of lead and opium, are the principal remedies whose modus operandi he discusses as being been found empirically to be the most successful. We shall notice some leading points in his therapeutics.

*Modus operandi of bleeding in thoracic inflammation.* This is most distinctly indicated in plethoric subjects, and its curative operation consists in the vacuum it causes in the vascular system; so soon as a vein is opened, the flow of the capillaries is rendered stronger while that in the larger veins is interrupted. This stronger flow from the venous capillaries is communicated to the arterial capillaries, and hence the vacuum is first felt in the arterial system. The left ventricle is thus enabled to receive, or rather suck in, more blood from the lungs through the pulmonary veins, and so the pulmonary capillaries are relieved from congestion. Further, the right ventricle can now transmit to the lungs the blood sent to it from the right auricle, and the vena cava, the root of the venous capillaries pours its blood more freely into the right auricle, and then the suction power of the heart augmented by the vacuum increases and is communicated to the whole capillary system of the organism. This ingenious explanation, Dr. Zimmermann argues, is supported by the mode in which the blood flows from the punctured vein, as first, the blood is forcibly projected because of the quantity collected below the bandage; then it flows with less impetus, and at last appears as if it would cease altogether, but after a while it is projected more forcibly, and when a few ounces have been taken, the impetus is so great as to render it not always an easy matter to stop it. This chain of phenomena is most observable in sanguineous apoplexy, and whenever it happens it is to be considered a good sign.

Fainting occurs at two stages of bleeding: the first, when only two or three ounces of blood have been taken; the second when the quantity amounts to twelve or eighteen ounces. Fainting in the first stage depends upon the action of the vacuum upon the left ventricle of the heart, and is a bad symptom, because it shows that the haustive power has not extended to the pulmonary veins and capillaries, and that the congestion there is permanent. Fainting in the second stage depends on anemia of the brain consequent on the depletion, and is a favorable symptom.

The re-establishment of the circulation in the pulmonary capillaries consequent on the vacuum caused by the abstraction of blood is followed by a restoration to healthy functions of the pulmonary tissue. The nerves act again normally: the air-cells relieved from pressure, become again permeable, and the ingress of oxygen to the blood is facilitated, while the pseudo-plasma is reabsorbed, provided it be recently effused, and have not undergone transformation. The albuminate of soda, which by its quality was a predisposing cause of inflammatory congestion, unless it have undergone change is still fit to carry on the progressive metamorphosis of the tissue; and if unfit, is acted on by the oxygen absorbed, and is thrown off through the kidneys and skin as an excretion. We need not discuss the modus operandi of topical depletion as the preceding views illustrate it sufficiently.

*The modus operandi of nitre in thoracic inflammation.* If, however, the congestion of the lungs is not recent, if effusion have taken place with morbid changes in the blood, in the capillaries, and in the organs themselves, bleeding is of secondary value, and other remedies must be adopted. Nitre and tartar emetic are the most generally and successfully adopted in Germany. The results of experiments show that the action of nitre on blood out of the body is to prevent its coagulation, to diminish the tendency of the blood-vesicles to unite, and to contract the membrane of the latter. Its chemical relations to fibrine as a solvent has been already stated. When taken into the stomach, it is absorbed into the circulation and excites both the capillaries and blood-vesicles to contract. It hinders the tendency of the fibrine to coagulate, and by rendering the effused plasma more soluble promotes its absorption. It also renders the absorption of oxygen into the blood more active, and so facilitates the decomposition of the pseudo-plasma and its excretion by the kidneys and skin, in the form of urate of ammonia, &c.

*Modus operandi of tartar emetic in thoracic inflammation.* Dr. Zimmermann instituted an experimental inquiry into the comportment of tartar emetic towards the blood when out of the body, and found that two grains added to one thousand of blood rendered the coagulation imperfect; and six grains caused the blood of a patient having the buffy coat to coagulate into a jelly-like mass, without any buff. Unlike nitre, it rendered the fibrine less disposed to decompose, and seemed to form with it rather an insoluble chemical compound. Unlike nitre also in its action on the blood-vesicles, it rendered them less contractile, and which examined under the microscope appeared large and expanded, the membrane being relaxed, and permitting the colouring matter to permeate it. Its modus operandi is therefore opposite to that of nitrate of potass, yet Dr. Zimmermann hesitates not to recommend them in combination. He is of opinion that the administration of tartar emetic alone without bleeding in extensive pneumonia and pleuritis is dangerous.

*The action of acetate of lead and opium in pneumonia.* Dr. Zimmermann says the great success of this combination (administered without bleeding) has been testified by so many and so high authorities, that there can be no doubt of its value as a remedy in pneumonia. He thinks the modus operandi of lead consists in its exciting action on the living fibre, and in its influence in arresting the progressive metamorphosis. His remarks amount however simply to this, that it is a good empirical remedy, is preferred by many to calomel, antimony, and the lancet, but that its modus operandi is unknown. Dr. Zimmermann gives no account of calomel as a remedy, although he administers it freely in practice: this is an important omission.

*Pathology of epidemic ophthalmia.* The remainder of the volume is occupied by a history of several cases of this disease, as it appeared in the garrison at Berlin. In each we have an analysis of the blood, and a daily analysis of the urine; and the whole is concluded by an extended inquiry into the pathology of the affection conducted with the ingenuity and research we have noticed as characteristic of his inquiries into the pathology of thoracic inflammations. We subjoin his summary of the condition of the blood in eight cases.

| Specific gravity of blood. | Solid contents. | Water. | Specific gravity of serum. | Solid contents. | Water. | Ratio of clot to serum. | Specific gravity of clot. | Solid contents. | Water. | Moist fibrine in grs. | Dry fibrine in grs. |
|---|---|---|---|---|---|---|---|---|---|---|---|
| 1053 | 207 | 793 | 1031 | 104·7 | 895·3 | 1·34 to 1 | 1087·2 | 319 | 781 | 6·4 | 2·25 |

The preceding results were obtained by his own method; the following by that of Andral and Gavarret. We would observe, however, that they are not the result of observation, but of calculation according to a formula laid down by Dr. Zimmermann, if we understand him rightly.

| Fixed residuum. | Water. | Fibrine. | Blood-vesicles. | Serum. | Fixed residuum of serum. |
|---|---|---|---|---|---|
| 207 | 793 | ·2·25 | 111·5 | 884·3 | 93·25 |

On a general review of our author we have to observe that his inquiries are a favorable example of the new researches in pathology to which the recent discoveries in organic chemistry must necessarily lead the physician. We think, however, that it would have been in better taste if more reference had been made to the views of Liebig. At least, Dr. Zimmermann should not have quoted the ipsissima verba of that chemist, without stating from whom he derived them, or indicating by inverted commas that the sentences were quotations. *Suum cuique* should be the motto, not less of the philosopher than the critic; but Dr. Zimmermann professes, and not without reason, to be both, and his authorship is therefore in this respect not to be approved.

